# A Balancing Act: The Viral–Host Battle over RNA Binding Proteins

**DOI:** 10.3390/v16030474

**Published:** 2024-03-20

**Authors:** Yahaira Bermudez, David Hatfield, Mandy Muller

**Affiliations:** Department of Microbiology, University of Massachusetts, Amherst, MA 01003, USA; ybermudez@umass.edu (Y.B.); dhatfield@umass.edu (D.H.)

**Keywords:** RNA binding proteins, protein–RNA interactions, RNA decay, RNA granules, viruses

## Abstract

A defining feature of a productive viral infection is the co-opting of host cell resources for viral replication. Despite the host repertoire of molecular functions and biological counter measures, viruses still subvert host defenses to take control of cellular factors such as RNA binding proteins (RBPs). RBPs are involved in virtually all steps of mRNA life, forming ribonucleoprotein complexes (mRNPs) in a highly ordered and regulated process to control RNA fate and stability in the cell. As such, the hallmark of the viral takeover of a cell is the reshaping of RNA fate to modulate host gene expression and evade immune responses by altering RBP interactions. Here, we provide an extensive review of work in this area, particularly on the duality of the formation of RNP complexes that can be either pro- or antiviral. Overall, in this review, we highlight the various ways viruses co-opt RBPs to regulate RNA stability and modulate the outcome of infection by gathering novel insights gained from research studies in this field.

## 1. Introduction

Viruses rely on intricate hijacking mechanisms to seize control of the host gene expression machinery for their own replication. Given the importance of RNA binding proteins (RBPs) in processes such as splicing, stability, localization, degradation, export, and translation, these proteins are at the center of this battle to control the gene expression resources [1,2,3]. Ribonucleoprotein complexes (RNPs) are assembled on mRNAs, and they reshape the fate of transcripts as these complexes are recruited via sequence motifs or sequence-independent secondary and tertiary structures [1,4]. Thus, the mRNA–protein interface can vary widely from forming stable RNP complexes to transient interactions. In infected cells, this is further complicated by the presence of viral proteins that may disrupt any number of these post-transcriptional processes. Furthermore, membrane-less organelles containing RNAs and RBPs are formed as compartmentalized densities that serve as important sites of host post-transcriptional regulation but can also be co-opted as viral replication factories [5,6,7]. While an extensive amount of work in the field has shown the importance of RBPs in the regulation of gene expression, and as a particular crux of targeting during viral infection [8,9,10,11], one important gap remains to be explored: how can the same RNP complexes be pro-viral in some contexts while antiviral in others? Studies portraying the impact of RNA–protein interactions during viral infection are an exciting avenue of research and will likely yield important information on transcript regulation. In this review, we will explore the complex interplay between RNA and proteins and their role(s) in RNA stability during viral infections, shedding light on new avenues to explore RNA fate and RBP modulation during viral infections in the future.

## 2. RNA Elements and Viral Infection

One strategy that viruses can use to redistribute gene expression resources is to induce broad RNA degradation events to support the needs of active viral translation. These RNA decay events are orchestrated directly by virally encoded endonucleases. Interestingly, recent work has begun to reveal RNA elements that are incorporated into select mRNAs that can drastically impact RNA stability in the face of these viral-induced decay events. Some of these RNA elements serve as target sites that recruit viral nucleases, while others can provide protective measures against viral-induced decay. For example, during Kaposi Sarcoma-Associated Herpesvirus (KSHV) lytic replication, the virally encoded endonuclease SOX causes widespread decay of the mRNA transcriptome [12]. However, a fraction of the transcriptome is spared from SOX-mediated degradation, which likely ensures the cell viability and proper progression of viral replication. Thus, it appears that SOX targets are carefully selected and in fact, the Gaglia group has recently mapped the SOX targeting motif, which is preferentially targeted during KSHV infection [13]. This targeting motif does not rely solely on sequence but rather on several key conserved residues alongside a potentially conserved structural motif [13,14]. Similarly, during Influenza A virus infection, the frameshift product PA-X functions as an endonuclease and contributes to a global viral-induced host shutoff. But how does PA-X specifically locate the right transcripts to degrade? Recently, Gaucherand et al. determined that PA-X targets GCUG tetramers in hairpin loops within transcripts for cleavage [15]. This specific element is widely abundant in the host genome but not in the viral genome, thus serving as a highly specific target to differentiate host mRNAs from viral ones [16]. Therefore, it emerges that using a viral nuclease with a wide range of host transcripts is an effective way to reallocate resources toward viral needs. Additionally, the extent of the mRNA target pool appears to be critical as a means to regulate viral infection. However, how the host fights back against the viral takeover and whether certain RNA elements could potentially resist viral nuclease targeting has recently been investigated [17,18,19]. In the context of KSHV infection, it was discovered that the transcript for Interleukin-6 (IL-6) encodes an RNA element within its 3’ UTR that can provide protection from SOX [17,18]. This RNA element of about 200 nucleotides was termed the Sox-Resistant Element (SRE) [18]. The SRE appears to fold into a stem-loop structure that modulates mRNAs’ susceptibility to the viral endonuclease and likely serves as a scaffold for the recruitment of RBPs [18]. Several RBPs were identified as specifically binding to this RNA element and forming a complex that prevents SOX-mediated decay [18,19]. Intriguingly, this “protective” element appears to dominate over the SOX targeting motif, as a transcript that contains both the targeting motif and the protective elements will remain unaffected by SOX [17,18,19]. This highlights that the stability and fate of an mRNA in the face of viral infection is controlled by the complex balance of its RBP landscape. Overall, the RNP environment on individual transcripts may serve as specific markers in the context of viral-induced decay and could provide clues to understand how viruses can distinguish between host and viral genes.

## 3. Host–Viral RBP Role during Viral Infections

Evolutionary pressure and the need to fit through their host translational machinery has driven viruses to mimic features of host transcripts to facilitate the hijacking of resources and escape immune detection [20,21,22]. By mimicking host features, especially altering their own transcripts, viruses also facilitate the hijacking of host RBPs and create RNP complexes that are indistinguishable from host RNPs [23,24]. For instance, during Dengue virus (DENV) infection, the receptor for activated C kinase 1 (RACK1)—a core component of the 40S ribosomal subunit—was shown to specifically interact with host factors SERBP1 and Vigilin to promote viral replication [25]. The authors denoted RACK1 as a platform to bind and recruit SERBP1 and Vigilin to the 40S ribosomal subunit to then collectively connect viral RNAs to the translation machinery to facilitate DENV infection [25]. Outside of infection, the ribosomal form of RACK1 has been demonstrated to help bridge mRNAs to polysomes, to recruit translational initiation factors, and contribute to translational quality control [25,26,27,28,29]. Yet, during DENV infection, RACK1 is co-opted to promote viral replication and acts as a binding platform at the 40S ribosomal subunit for the recruitment of DENV host-dependency factors [25]. Therefore, by hijacking key host RBPs, viruses ensure the progression of infection [30,31,32]. However, not all cellular RBPs favor viral replication, and mimicking host mRNAs can be a double-edge sword as some RBPs can have potent antiviral effects. For example, the host factor Shiftless (SHFL) is a RBP that has been shown to restrict the gene expression of a number of viruses from inhibiting HIV frameshifts to manipulating cytoplasmic RNP granules [11,33,34]. Another example is the RNA binding protein polypyrimidine tract-binding protein 1 (PTBP1), which was shown by Qin et al. to target and degrade the viral nucleocapsid (N) protein by activating the MARCH8-NDP52 autophagosome pathway in porcine epidemic diarrhea virus (PEDV) [35]. Collectively, these studies illustrate the profound impact cellular RBPs can have on the outcome of an infection and the duality in their functions. By further understanding the mechanisms by which cellular factors modulate processes in a pro-viral or antiviral way, we will continue to better decipher the complex landscape of RNA–protein interactions during viral infections.

## 4. RBP Modulation of RNA Stability in Subcellular Localizations during Viral Infections

The physical separation between the processes of transcription and translation enables RNA–protein complexes to adapt and diversify throughout the life of an mRNA, a factor that is contingent on its subcellular localization. However, during viral infections, viral RNAs (vRNAs) compete with cellular RNAs to take over resources from the cell. Viruses, by hijacking cellular RBPs, can disrupt the RBPs’ normal localization and their function. To date, several studies have investigated the mechanisms behind RBP re-localization in response to cellular environmental changes, particularly during viral infection [36,37,38,39]. In this section, we will review how RBPs regulate RNA stability between cellular compartments during viral infections.

### 4.1. Nuclear and Cytoplasmic Regulation

RNA fate is highly linked to its subcellular localization, a process largely driven by the pattern of RBPs on transcripts [40,41,42]. In the context of viral infection, the dynamic process of nuclear–cytoplasmic shuttling can be drastically altered [30,43]. For example, in the context of gamma-herpesvirus infection where host shutoff leads to widespread mRNA decay, many RBPs could potentially become a target to facilitate viral replication [43]. Using TMT labelling coupled to LC/MS-MS, Gilbertson et al. were able to track the subcellular localization of RBPs and showed that numerous RNA binding proteins undergo a change in their localization in response to viral-induced decay [43]. Given the scale of RNA decay triggered by these viruses, it is perhaps unsurprising that many RBPs would lose their target mRNA and be released, many of them finding their way back to the nucleus [43]. This potentially creates a measurable cellular response “sensing” of RNA decay in the cytoplasm, and the message can be relayed to the nucleus through the shuttling of RBPs [43,44,45,46,47]. According to Gilbertson et al., this creates a feedback mechanism, akin to informing the transcription machinery of the state of RNA decay in the cytoplasm and resulting in a halt of host transcription [43,47]. Therefore, trafficking of RBPs between cellular compartments during events of stress such as viral-induced RNA decay could be fundamental to relaying information about cellular mRNA abundance within the cell [41,43,47]. Another study by Garcia-Moreno et al. observed similar dynamics of RBP in response to Sindbis Virus (SINV) infection [30]. The authors used a system-wide approach known as RNA-interactome capture (RIC) to determine the distribution of RBPs in cells infected with SINV [30]. In the context of drastic loss of cellular RNAs and high abundance of viral RNAs, they observed a remodeling of the RBP interactome [30]. In particular, it was observed that many key gene expression regulators such as UPF1 (helicase, nonsense-mediated decay pathway), SRPK1 (alternative splicing, RNA export and stability), GEMIN5 (mediates assembly of small nuclear RNPs), TRIM25 and TRIM56 (E3 ubiquitin ligases), PPIA (regulation, signaling, apoptosis), and FAM98A (form the tRNA ligase complex) were re-localized to viral factories [30]. These proteins were observed to colocalize with viral RNAs, suggesting a role in facilitating viral replication during SINV infection. However, it remains unclear what the cell response is to drastic loss of host RNA and RBP re-localization during SINV infection and how remodeling of viral–host protein interactions could contribute to alter RBP function and localization during SINV infection. 

It is becoming clear that RBP function and localization is pivotal during viral infections and can help viruses co-opt resources. Many questions continue to mount surrounding the mechanisms that regulate the shuttling of RBPs during viral infection, especially during viral-induced RNA decay. With the advent of novel protein labeling methods, we anticipate that we will learn more about the dynamics of these processes in the coming years. 

### 4.2. Nuclear Export

**Figure 1:**
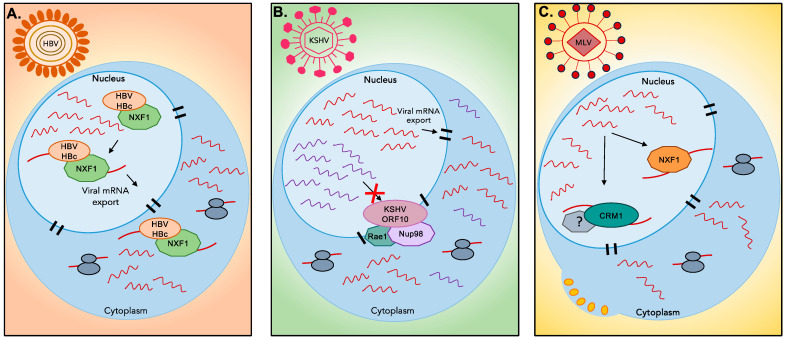
Viral manipulation of nuclear export pathways: (**A**) HBV viral protein HBc binds export proteins NXF1 to facilitate export of viral transcripts (red). (**B**) KSHV viral protein ORF10 binds export proteins NXF1-NXT1 to block export of cellular transcripts (purple) promoting export of viral transcripts (red). (**C**) MLV viral transcripts interact with NXF1 protein to promote their transport from the nucleus into the translation machinery, while other viral transcripts interact with CRM1 which marks them for further viral packaging.

Once mature, mRNAs are transported to the cytoplasm through nuclear pore complexes (NPCs) embedded in the nuclear envelope [48,49,50,51]. Transport of mRNAs is mediated by mRNP complexes composed of shuttling proteins, commonly the NXF1-based bulk export, and a more specialized CRM1-based export for unspliced mRNA [49,52]. Several studies have shown that viruses target the nuclear export machinery, which results in the downregulation of host gene expression and reduction in host antiviral responses [51,53,54,55]. For instance, Hepatitis B Virus (HBV) core protein HBc interacts with NXF1 [56]. This interaction is suggested to mediate the shuttling of the Hepatitis B core/capsid protein (HBc)–NXF1 complex, and facilitate the export of HBV transcripts (Figure 1) [56]. During KSHV infection, Gong et al. identified the interaction of the viral protein ORF10 with nuclear pore proteins (Nup98) and export factors (Rae1), which results in blocked mRNA export and the nuclear accumulation of transcripts (Figure 1) [55]. However, this export inhibition is not total but instead relies on transcript selectivity based on an unidentified RNA element located at their 3’UTR [55], which likely ensures proper export of viral mRNAs. This inhibition contributes to the global “host shutoff” phenotype induced by herpesviruses and helps with ensuring easier access to the translational machinery for viral transcripts [55,57]. Meanwhile, in Murine Leukemia Virus (MLV), both nuclear export routes (NXF1 and CRM1) are proposed to be exploited by the virus [58]. Mougel et al. show that MLV full-length RNA interacts with NXF1, allowing export of viral RNAs destined to undergo translation (Figure 1) [58]. Simultaneously, export of MLV RNA by CRM1 marked transcripts for viral packaging in the cytoplasm [58]. The use of these two export pathways by MLV highlights the ability of the viral RNA to assemble two different mRNP complexes and control RNA fate. Overall, many of the components of the nuclear export pathway have been identified as viral targets but the precise mechanisms by which their hijacking is coordinated are still not well understood. Ongoing and future studies will likely shed light on the processes that regulate the fate of viral and cellular mRNAs through the recruitment of RNA–protein complexes. 

## 5. RNA Granules: A Nexus in the Viral–Host Struggle over RNA

RNA granules are biomolecular condensates of RNA and protein that exist within the cytoplasm. While these densities are often described to undergo liquid–liquid phase separation (LLPS), the story is more complex [59]. A better description of LLPS helps to better illustrate what these foci are: sequestered conglomerates of biomolecules that are separated from the surrounding cytoplasmic matrix without any phospholipid barrier [59]. However, this distinction can be quite difficult to define in such a dynamic system [60,61,62]. Granules are not simply defined as viscous liquids but are more precisely described as viscoelastic densities [63,64,65,66,67,68]. This distinction implies a duality and fluctuation of solid and liquid elements, and active exchange with the environment. Two classifications of cytoplasmic granules have emerged as important regulatory mechanisms within cells and both heavily rely on complex and dynamic interactions between RBP and RNA: processing bodies (P-bodies) and stress granules (SGs) [69,70]. P-bodies are often comprised of mRNA and RBPs such as DDX6, ET-4, PAT1B, LSM14A, EDC4, DCP1/DCP2, and CPEB1 [71,72,73,74,75,76,77,78,79,80,81,82]. Along with ribonucleic acids, SGs often contain proteins such as G3BP1/G3BP2, TIA-1, TIAR, Atx2, eIF2, eIF3, eIF4A, eIF4B, eIF4E, eIF4G, and eIF5 [69,82,83,84,85,86,87,88,89]. Some granules, such as P-bodies, are constitutively expressed in cells, and treatment with translation elongation inhibitors, like cycloheximide, will lead to cytoplasmic loss of the granules [70,71,90]. Conversely, stress granule formation must be induced by treatment with a translation terminator such as puromycin [70,91] or via cellular stressors like viral agents [5]. P-bodies are thought to be sites of RNA metabolism and regulation within the cytoplasm and have been implicated in mRNA decay, translation repression, nonsense-mediated decay (NMD), as well as mRNA silencing. They have commonly been identified as sites of mRNA decay due to several constituents’ ties to transcript degradation, namely DDX6, DCP1, DCP2, Xrn1, EDC3, and EDC4, among others. However, they can also serve as compartments to temporarily sequester some transcripts from the translation pool to control pathway activation/deactivation or for energetic conservation [70]. On the other hand, it has been well characterized that SGs form upon viral infection, and these cytoplasmic granules are often targeted for downregulation by viruses.

**Figure 2:**
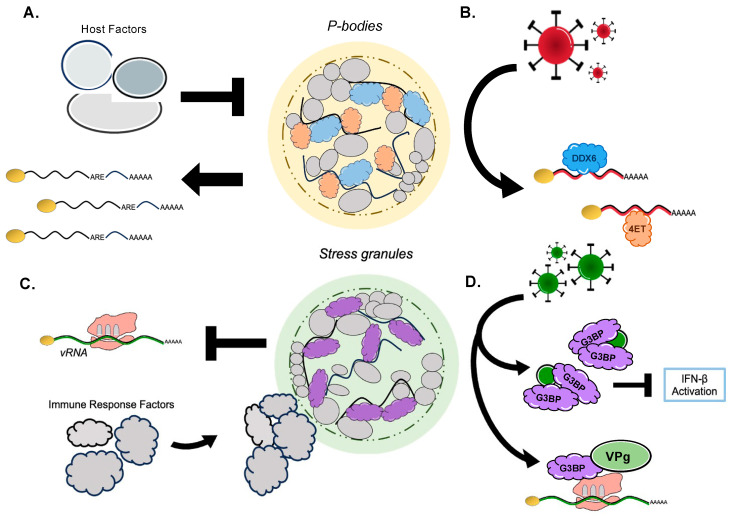
RNA granule functionality in the context of viral infection. (**A**) Host factors, such as SHFL or NBDY, result in P-body loss, which can impact the availability of ARE-mRNA transcript levels. (**B**) Viral agents like KSHV, HCV, WNV, and EV71 recruit P-body RBPs to promote viral RNA stability and translation. (**C**) Stress granule induction helps to slow viral translation, while serving as a scaffold to mount an immune response and combat infection. (**D**) Viruses, such as SARS-CoV-2, MNV, and HuNoV, have been observed to re- purpose G3BP1 leading to a blockage for certain anti-viral pathways. G3BP1 may also be repurposed during infection to promote stability for viral translation complexes.

Due to their dynamic nature, diversity in composition, and temporal/environmental sensitivity, RNA granules’ functionality varies broadly from mRNA storage, degradation, and triage to assisting in cellular processes such as energetic conservation, the stress response, or the inflammatory response. This multitude of processes can readily be taken advantage of by viruses. Viruses and the host wage constant battles over the formation and regulation of RNA granules by manipulating RBPs, in a means to ultimately influence gene expression and lead to the arrest of certain cellular pathways to control the fate of RNA (Figure 2).

### 5.1. Viral Factors Exercise Control over P-Body RBPs to Promote Viral Replication

Given their extensive role in regulating mRNA fate, P-bodies are prime targets for viruses that need to co-opt these pathways for their own replication. It has previously been shown that viruses such as Kaposi Sarcoma-Associated Herpesvirus (KSHV), Hepatitis C Virus (HCV), and West Nile virus (WNV) all utilize P-body constituents such as DDX6, Lsm-1, DDX3, Ago2, Xrn1, and others to promote viral RNA and protein stability [8,92,93,94,95,96,97,98,99]. Recently, an investigation of Enterovirus 71 (EV71) showed how the virus induces a loss of P-bodies within human cells, which Fan et al. demonstrated stems from blocking the formation of de novo P-bodies [100]. The group further determined that certain scaffold proteins (i.e., DDX6 and 4E-T, among others) of P-bodies affected viral mRNA transcript levels [100]. The absence of DDX6 and 4E-T leads to decreased viral transcript levels compared to when both proteins were expressed [100]. Thus, they hypothesized that virally induced P-body loss reallocated and repurposed key P-body components for RNA stability and expression [100]. The group’s final model highlighted that EV71’s protease 2C facilitates host RBP interactions with viral mRNA to promote viral gene expression, which ultimately leads to the blockage in the formation of P-bodies [100]. It emerges that viruses are masters at seizing control of P-bodies through their interactions with host RBPs and use them to promote virion production. This also potentially suggests that the regulation of host gene expression could be suppressed to further remodel the host environment, making it more favorable for viral replication. 

### 5.2. Hosts Manipulate P-Body RBPs to Alter RNA Availability/Degradation to Combat Viral Infection

It has been widely reported that P-bodies often house transcripts with AU-rich elements (AREs), located in their 3’ UTR for the purposes of degradation or translational repression [101,102,103,104,105,106]. Many ARE-bearing transcripts encode for chemokines and cytokines [107,108]. Several groups have demonstrated that following P-body loss, an increase in ARE-mRNAs follows [101,102,103,104,105,106]. These results suggest that a decrease in P-body counts may in fact play an antiviral role with the increased expression of these effectors. However, it remains unclear to what extent does the host or virus individually contribute to the loss of P-bodies during infection. Are P-bodies altered to stimulate the host inflammatory response to mount an antiviral response or to enhance an environmental favorability for viral agents? A host RBP that we mentioned previously in this review—Shiftless (SHFL)—is a broad-acting and potent antiviral factor [11,33,109,110,111,112,113,114] with known roles in regulating cytoplasmic granules. In the context of KSHV, SHFL has been shown to restrict P-body foci [11]. The exact subcellular mechanism through which SHFL accomplishes this P-body loss remains unclear. SHFL localizes to P-bodies [115] and its overexpression causes the loss of P-body formation [11]. The SHFL-mediated depletion of P-bodies could subsequently alter the expression levels of certain ARE-mRNAs that are often included in these granules, which likely contribute to SHFL overall ability to restrict viral agents. Outside of the context of infection, cellular loss of P-bodies typically occurs through two methods: 1. The depletion of core protein components, or 2. The expression/phosphorylation of the 68-amino-acid microprotein—the non-annotated P-body dissociating polypeptide (or “NBDY”) [69,86,116]. Losses of certain proteins such as LSm14a [73,77], DDX6 [75,77], or EDC4 have all been shown to lead to losses in P-bodies [72]. Otherwise, the expression and phosphorylation of the polypeptide NBDY has been shown to cause the loss of P-bodies, by potentially disrupting the electrostatic networks of these granules [117,118]. However, it has also been posited that P-body regulation may also release many decapping and endonucleolytic enzymes, leading to a translation suppression [119]. Therefore, it is possible that during viral infection, the host attempts to control the cytoplasmic gene expression environment by disassembling P-bodies. Viral agents often seek control over P-body RBPs as a tool to influence RNA expression within the cell. All the while, the host attempts to antagonize viral replication through a similar method: utilizing P-body-associated RBPs to alter RNA expression for antiviral pathways.

### 5.3. Viruses Influence Stress Granule RBPs to Suppress Host Immune Response Transcripts and Positively Regulate Viral RNA Fate

Numerous viruses have been shown to affect SGs: Japanese Encephalitis Virus (JEV) has been shown to alter the localization of SG marker G3BP1 [120]; Dengue virus (DENV), West Nile virus (WNV), Murine Respirovirus (SeV), and the Zika virus (ZIKV) have all been shown to utilize viral biomolecules to sequester SG critical proteins to block granule formation [9,121,122]. Recently, the betacoronavirus, severe acute respiratory syndrome coronavirus 2 (SARS-CoV-2), has been shown to have a similar effect on SG abundance and the re-purposing of the foci’s components [123]. The SARS-CoV-2 nucleocapsid protein (NP) has also been found to interact with the structurally critical SG proteins G3BP1/G3BP2 [123]. Liu et al. observed that this NP-G3BP interaction limits the degree to which SGs can form in response to viral infection, which in turn limits the host’s ability to stall translation and slow viral replication [123]. The group went on to observe that SARS-CoV-2’s NP sequestration of G3BPs not only resulted in the loss of SGs but also in the downregulation of IFN-β transcript levels and the RIG-I pathway (a critical piece of the innate immune response) as a whole [123]. This differs from a normal immune response, where G3BP1 typically influences the RIG-I pathway and IRF3 becomes phosphorylated, ultimately causing an increase in IFN-β transcription [124]. The researchers were then able to demonstrate how this virus exercises control over SG RBPs, such as G3BP1, to ultimately increase the propensity for viral mRNAs and translation. Given the increase in viral mRNAs in the absence of G3BP1, the authors concluded that the NP-G3BP1 interactions could help facilitate viral replication [124]. A genomic intermediate of the SARS-CoV-2 genome appears to exist as dsDNA, which has an observable affinity for G3BP1; mechanically, this was hypothesized to be how the virus isolates this RBP from the host’s antiviral system [124]. Additionally, this SG component has been shown to be necessary for both murine norovirus (MNV) replication as well as Norwalk norovirus replication (a replicon used to model human norovirus, HuNoV) [125]. Following the knockout of G3BP1 within cells, both MNV and HuNoV were observed to have significantly low transcript levels and were no longer tied to cytotoxic outcomes; cells were seen to be virally resistant [125]. Interestingly, Hosmillo et al. were able to uncover that G3BP1 RNA binding domains directly impacted viral replication; the loss of these domains resulted in less viral yield [125]. In addition, Hosmillo and colleagues discovered that G3BP1 interacts with the viral protein VPg and helps facilitate the loading of ribosomes and polysomes onto norovirus RNA [125]. It thus emerges that SGs are fascinating focal points for where the host and virus compete over RBPs to gain an advantage over the other.

### 5.4. Host Agents Orchestrate Stress Granule RBPs to Effectively Quench Viral Replication

Stress granules (SGs) have been widely characterized as antiviral subcellular compartments. In the case of the α-coronavirus porcine epidemic diarrhea virus (PEDV), SGs form and eventually are lost toward the latter stages (i.e., around 36 h) of infection [126]. In the study, Sun et al. identified that the overexpression of G3BP1 resulted in significantly decreased levels of viral mRNA [126]. They further discovered that PEDV encodes a protease that cleaves G3BP1 in the late stages of infection, which causes this loss [126]. They were able to pinpoint the specific cleavage sites that viral caspase-8 targets down to two aspartic acid residues [126]. Interestingly, robust SGs with cleavage-resistant G3BP1 stringently limited viral transcripts as well as overall viral titers, indicating that SGs can efficiently limit viral transcription and translation [126]. Studies like these demonstrate the capability and antiviral functionality of SGs and how the host attempts to use RBPs like G3BP1 to restrict viral agents. 

### 5.5. Granule Functionality/Effects Are Convoluted; Both Virus and Host Wield SG RBPs for Their Own Benefit

The above sections detailing the great virus and host struggle over P-body and SG RBPs already outline how ambiguous the functionality of granules can be. One other fascinating viral case study that depicts this murkiness can be seen in the context of Viral Hemorrhagic Septicemia Virus (VHSV), a member of Rhabdoviridae, which appears to trigger the formation of stress granules [127]. Interestingly, G3BP1 was shown to be redistributed to viral replication complexes and proven to be essential for efficient virion production [127]. Even though viral infection results in SG formation, VHSV still influences G3BP1 to facilitate its own replication [127]. However, SGs also seem to play an antiviral role within the system. G3BP1 knockdown led to an increase in IFN signaling and a decrease in VHSV protein levels and viral titers [127]. This suggests that G3BP1 and SGs can limit viral replication to an extent, likely via translational arrest and sequestration [127]. VHSV utilizes SG RBPs such as G3BP1 to enhance replication, yet the host also exercises some control over these RBPs to simultaneously limit viral replication [127]. This case of viral infection and SG dynamics captures the convoluted subcellular environment and depicts how RNA granules exist at the axis where pro-viral and pro-host states teeter. Have viral agents robustly conquered this host defense and adapted to largely benefit from granules? Will host mechanisms adapt or can they be shaped to repurpose the likes of P-bodies and SGs to effectively combat viral replication?

## 6. Conclusions and Future Perspectives

RNA binding proteins comprise the subcellular foundation for host survival and viral replicative strategies. While this review covers many of the most recent findings of how both sides of this conflict attempt to take advantage of these proteins, new studies continue to unveil different viral case studies and mechanisms of exploit. mRNP complexes present an exciting avenue of study to better understand viral replication and takeover. As proper gene expression requires fine-tuned RNA processing, this multifaceted quality control system helps to regulate the cell homeostasis. It can prove to be disastrous when certain viral agents target one juncture of RNA processing and critical information is lost. Certain host factors, such as NXF1 and CRM1, critically regulate nuclear egress of transcripts, which can largely impact host survival dependent upon whether the virus or host controls these export pathways. Interestingly, RBPs can also help regulate the dynamics of certain RNA granules, which can significantly impact gene expression. Through P-bodies and stress granules, RBPs influence mRNA sequestration, decay, and release into the translational pool. 

Furthermore, from the studies reviewed here, we can continue to interrogate how cellular pathways are affected by viral takeover, but also how the host responds to the viral attack. As mRNA abundance and availability can be drastically altered, cellular feedback response pathways can potentially serve to relay stress signals during viral infection. This area is still ripe for further study since it is still unclear which cellular factors are involved and to what extent communication is carried out in the cell between cellular compartments. Moreover, more studies to discern RNA–protein interactions when RBP expression levels are altered during viral infections are imperative to shed light onto the unknown biological significance of RBP activity. This will reveal crucial information on RBP localization, RBP multifunctionality, and their response to target availability. Studies in these areas could prove exceptionally fruitful, providing a deeper understanding of these processes, which could lead to the discovery of new antiviral targets and the development of therapeutic agents.

Regarding the RBPs associated with RNA granules, many factors and mechanisms are yet to be discovered. For viral agents, such as EV71, that lead to P-body loss, it remains to be seen mechanistically how the virus utilizes and recruits RBPs like DDX6 to provide stability and promote viral gene expression. Further characterization also ought to be conducted on exactly how the host wields P-bodies and their biomolecules specifically to alter its own gene expression to ultimately combat viral gene expression. For stress granule RBPs, how prevalent is this viral hijacking strategy? What other viral agents have evolved mechanisms to disrupt SG formation for purposes other than a lack of transcript sequestration? Overall, the characterization of RNA granule functionality during viral infection has emerged as an exciting frontier for better understanding virus–host interactions at the subcellular level.
